# Development of Autoimmune Diseases Among Children With Pediatric Acute-Onset Neuropsychiatric Syndrome

**DOI:** 10.1001/jamanetworkopen.2024.21688

**Published:** 2024-07-30

**Authors:** Meiqian Ma, Erin E. Masterson, Jaynelle Gao, Hannah Karpel, Avis Chan, Rajdeep Pooni, Jesse Sandberg, Erika Rubesova, Bahare Farhadian, Theresa Willet, Yuhuan Xie, Paula Tran, Melissa Silverman, Margo Thienemann, Elizabeth Mellins, Jennifer Frankovich

**Affiliations:** 1 Division of Allergy, Immunology, & Rheumatology, Department of Pediatrics, Stanford University School of Medicine, Palo Alto, California; 2 Stanford Immune Behavioral Health Clinic and Research Program at Lucile Packard Children’s Hospital, Palo Alto, California; 3 Department of Environmental & Occupational Health Sciences, School of Public Health, University of Washington, Seattle; 4 Pediatric Division of Radiology, Stanford University School of Medicine, Palo Alto, California; 5 Division of Child & Adolescent Psychiatry, Department of Psychiatry, Stanford University School of Medicine, Palo Alto, California; 6 Department of Pediatrics, Program in Immunology, Stanford University School of Medicine, Palo Alto, California

## Abstract

**Importance:**

Epidemiologic studies indicate a high rate of autoimmune conditions among patients with obsessive-complusive disorder and other psychiatric conditions. Furthering the understanding of the inflammatory diatheses of psychiatric conditions may open doors to new treatment paradigms for psychiatric disorders.

**Objectives:**

To evaluate whether pediatric acute-onset neuropsychiatric syndrome (PANS) is associated with an inflammatory diathesis by assessing signs of immune activation and vasculopathy during a psychiatric symptom exacerbation (flare), estimating the risk of developing arthritis and other autoimmune diseases, and characterizing subtypes of arthritis.

**Design, Setting, and Participants:**

This retrospective cohort study used longitudinal clinical data on 193 consecutive patients with PANS followed up within the Stanford Immune Behavioral Health Clinic from September 1, 2012, to December 31, 2021.

**Main Outcomes and Measures:**

Medical records were reviewed, and a predefined set of immune markers that were measured during a flare and the features and imaging findings of arthritis and other autoimmune diseases were collected. Immune activation markers included (1) autoimmunity signs (antinuclear antibody, antihistone antibody, antithyroglobulin antibody, C1q binding assay, and complement levels [C3 and C4]); (2) immune dysregulation or inflammation signs (leukopenia, thrombocytosis, C-reactive protein, and erythrocyte sedimentation rate); and (3) vasculopathy signs (livedo reticularis, periungual redness and swelling, abnormally prominent onychodermal band, palatal petechiae, high von Willebrand factor antigen, and high d-dimer). Last, the cumulative risk of developing arthritis and autoimmune diseases was estimated using product limit (Kaplan-Meier) survival probability.

**Results:**

The study included data from 193 children (112 boys [58.0%]) who had PANS at a mean (SD) age of 7.5 (3.5) years. They were followed up for a mean (SD) of 4.0 (2.1) years. Among those tested for immune activation markers, 54.2% (97 of 179) had nonspecific markers of autoimmunity, 12.0% (22 of 184) had nonspecific signs of immune dysregulation or inflammation, and 35.8% (69 of 193) had signs of vasculopathy. By 14 years of age, the estimated cumulative incidence of arthritis was 28.3% (95% CI, 20.8%-36.3%), and the estimated cumulative incidence of another autoimmune disease was 7.5% (95% CI, 4.0%-12.4%). Novel findings in the subgroup with arthritis include joint capsule thickening (55.0% [22 of 40]), distal interphalangeal joint tenderness (81.8% [45 of 55]), and spinous process tenderness (80.0% [44 of 55]). Among the 55 patients with arthritis, the most common subtypes of arthritis included enthesitis-related arthritis (37 [67.3%]), spondyloarthritis (27 [49.1%]), and psoriatic arthritis (10 [18.2%]).

**Conclusions and Relevance:**

This study found that patients with PANS show signs of immune activation and vasculopathy during psychiatric symptom flares and have an increased risk of developing arthritis and other autoimmune diseases compared with the general pediatric population. The most common arthritis subtype was enthesitis-related arthritis. These findings suggest that PANS may be part of a multisystem inflammatory condition rather than an isolated psychiatric or neuroinflammatory disorder.

## Key Points

**Question:**

Do clinical signs indicate that pediatric acute-onset neuropsychiatric syndrome (PANS) is a systemic inflammatory condition?

**Findings:**

In this cohort study of 193 children with PANS, there was a high frequency of nonspecific autoimmune markers (54.2%), immune dysregulation and inflammation markers (12.0%), and vasculopathy markers (35.8%) and a high estimated risk of developing arthritis and an additional autoimmune disease (28.3% and 7.5%, respectively) at 14 years of age. Those with arthritis often had joint capsule thickening (55.0%); common and unique physical examination findings included distal interphalangeal joint tenderness and spinous processes tenderness.

**Meaning:**

This study suggests that some children with a diagnosis of PANS show signs of an underlying inflammatory process and are at risk of developing autoimmune disease.

## Teaser

This cohort study evaluates whether pediatric acute-onset neuropsychiatric syndrome is associated with inflammation by assessing signs of immune activation and vasculopathy during a psychiatric symptom flare, estimating risk of arthritis and other autoimmune diseases, and characterizing arthritis subtypes.

## Introduction

Pediatric acute-onset neuropsychiatric syndrome (PANS) is characterized by abrupt-onset obsessive-compulsive symptoms plus other abrupt-onset neuropsychiatric symptoms (sleep disruption, urinary changes, cognitive function loss, behavior deterioration, emotional lability, and irritability) causing significant patient, family, or caregiver distress.^1,2,3^ Pediatric acute-onset neuropsychiatric syndrome follows a relapsing-remitting course of flares (abrupt psychiatric symptom deteriorations) between periods of relative symptom quiescence.^4,5^ Classification of a pediatric autoimmune neuropsychiatric disorders associated with streptococcal infections (PANDAS) requires a temporal association with group A streptococcal (GAS) infection.^6^ The more general term, PANS, describes similar symptoms as PANDAS but is agnostic to the preceding infection^7^ (eAppendix 1 in Supplement 1). Provisional management recommendations for PANS include (1) clearing an infection if present, (2) anti-inflammatory treatments, and (3) standard psychiatric care.^8,9,10,11,12,13^ Anti-inflammatory treatments include intravenous immunoglobulin, corticosteroids, and nonsteroidal anti-inflammatory drugs, but randomized placebo-controlled trials are lacking.^4,5,13,14^

Emerging evidence shows a role for adaptive and innate immune systems in PANS and PANDAS,^7^ likely involving basal ganglia inflammation. Imaging demonstrates basal ganglia swelling in the acute stage,^15^ microglia activation,^16^ and microstructural changes.^17,18^ Xu and colleagues^19^ identified autoantibodies targeting and altering cholinergic interneuron function in the basal ganglia. Patients with PANS or PANDAS have movements during rapid eye movement sleep—a factor associated with other basal ganglia disorders, including Parkinson disease.^20,21,22,23,24^ Chain and colleagues^25^ found subsets of autoantibodies distinguishing patients with PANDAS from controls. Animal models of PANDAS demonstrate an adaptive immune response, involving autoantibodies and Th17 cells, leading to central nervous system pathologic findings, including neurovascular damage affecting the basal ganglia.^26,27^ Rahman and colleagues^28^ reported that proinflammatory monocytes were more elevated during flare periods vs recovery periods; both periods had higher levels than controls. Approximately one-third of patients with PANS undergoing lumbar puncture have elevated levels of cerebrospinal fluid protein and/or elevated levels of the albumin quotient, which may be a nonspecific marker of neuroinflammation. These findings support an inflammatory diathesis in an immunogenetically susceptible host.

We postulate that postinfectious neuroinflammation of PANS or PANDAS might be accompanied by or associated with systemic inflammation, as is seen in acute rheumatic fever, where patients often develop obsessive-compulsive disorder (OCD), emotional lability, involuntary movements, arthritis, vasculitis, carditis, and other inflammatory signs.^29,30^ Given the suspected inflammatory underpinnings of PANS, we aimed to evaluate signs of immune activation and vasculopathy during flares and estimate the risk of developing arthritis and other autoimmune diseases among children with PANS.

## Methods

### Study Design and Participants

This retrospective cohort study analyzes observational longitudinal clinical and laboratory data from pediatric patients followed up in the multidisciplinary Stanford Immune Behavioral Health Clinic. This study protocol was approved by the Stanford Human Participants institutional review board and covers all patients in this study. Immune Behavioral Health Clinic clinicians cared for all patients. Written parental consent and assent were obtained as part of our prospective biomarker study (protocol number 26922). The Strengthening the Reporting of Observational Studies in Epidemiology (STROBE) reporting guideline was followed.

We evaluated 420 patients’ electronic medical records from September 1, 2012, to December 31, 2021, to select our analytic sample of 193 patients who met PANS criteria^7,31^ as determined by child psychiatrists (Y.X., P.T., M.S., and M.T.) and were followed up for at least 3 months in the clinic (eAppendix 1 and eMethods in Supplement 1).

Between December 2, 2019, and March 19, 2022, we reviewed electronic medical record histories, collateral records, and parent-response questionnaires to abstract demographic data, including self-reported race and ethnicity, laboratory test results, diagnoses, physical examination results, and findings from musculoskeletal imaging. All data were entered into REDCap. Race and ethnicity categories used were those from the Office of Management and Budget 1997 categories based on National Center for Education Statistics. We assessed race and ethnicity in this study for a better understanding of whether the results are generalizable to a specific racial and/or ethnic population.

### Measurements

#### Laboratory and Physical Signs of Immune Activation

We evaluated immune activation signs based on a predetermined set of laboratory and physical examination findings indicative of (1) autoimmunity (antinuclear antibody [ANA; titer ≥1:80], antihistone antibody [high or normal], antithyroglobulin antibody [high or normal], C1q binding assay [high or normal], and complement levels [C3 (low or normal) and C4 (low or normal)]); (2) immune dysregulation or inflammation (indication of leukopenia [white blood cell count <4000 cells/µL (to convert to ×10^9^/L, multiply by 0.001)], thrombocytosis [platelet count >400 × 10^3^ cells/µL (to convert to ×10^9^/L, multiply by 1.0)], C-reactive protein [high or normal], and erythrocyte sedimentation rate [high or normal]); and (3) vasculopathy (livedo reticularis, periungual redness and swelling, onychodermal band [abnormally prominent or normal; eFigure 1 in Supplement 1], palatal petechiae [present or normal], von Willebrand factor antigen [high or normal], and d-dimer [high or normal]). Laboratory test results came from Stanford and other clinical laboratories. Each laboratory’s reference ranges were used to determine abnormalities. We report the first laboratory data collected during a flare episode and the patient not receiving immunomodulatory therapy. Laboratory data were collected during the same flare episode for 47.2% of patients (91 of 193), across 2 episodes for 29.5% of patients (57 of 193), and across 3 or more episodes for 23.3% of patients (45 of 193). Positive physical examination findings were those recorded at least twice prior to immunomodulation.

#### Arthritis Classification

Rheumatologists’ records and patient questionnaire responses were used to assess joint pain, stiffness, swelling, warmth, redness, entheseal or joint tenderness, nail pitting, signs and symptoms of inflammatory back pain (by criteria of Calin et al^32^), and musculoskeletal imaging findings (eAppendix 2A in Supplement 1). Pediatric rheumatologists (M.M. or J.F.) classified patients with arthritis if they had (1) pain and joint effusion (lasting >6 weeks); (2) pain and 2 or more of the following: limited or painful range of motion, tenderness, or warmth (lasting >6 weeks); or (3) joint tenderness and ultrasonographic confirmation of arthritis. The first 2 categories meet American College of Rheumatology criteria for juvenile rheumatoid arthritis.^33^ Patients were further classified by International League of Associations for Rheumatology (ILAR)^34^ and/or Assessment of SpondyloArthritis international Society (ASAS)^35,36^ criteria. Patients who were 16 years or older at diagnosis were classified using ILAR criteria if their symptoms began before 16 years of age. We applied ILAR enthesitis-related arthritis (ERA) criteria to patients 16 years of age or older because there are no classification criteria for ERA in adults.

Musculoskeletal ultrasonography of hands, wrists, feet, and ankles were obtained if (1) pain and stiffness persisted despite arthritis medication; (2) suspicion for arthritis was high based on history, but physical examination findings were not definitive; and/or (3) the history or physical examination was incomplete due to psychiatric symptoms. Magnetic resonance imaging was performed to assess for inflammation involving the pelvis, spine, and temporomandibular joint, which are difficult to assess with ultrasonography. All musculoskeletal ultrasonographic procedures were conducted at Stanford and interpreted by a board-certified radiologist (E.R.) with expertise in musculoskeletal imaging (eAppendix 3 in Supplement 1).

#### Autoimmune Disease and Other Immunologic Disease Classifications

Evaluations by pediatric subspecialists were the basis for assigning diagnoses of autoimmune thyroiditis, psoriasis, inflammatory bowel disease (including Crohn disease, ulcerative colitis, and common variable immunodeficiency-related inflammatory bowel disease), celiac disease, Behcet disease, systemic lupus erythematosus, or type 1 diabetes (eAppendix 2B and C in Supplement 1). The category *other immunologic disease* includes primary immunodeficiency; chronic urticaria; periodic fever, aphthous stomatitis, pharyngitis, and adenitis; and eosinophilic esophagitis.

### Statistical Analysis

We report descriptive statistics for demographic information, clinical characteristics, signs of immune activation, and arthritis and autoimmune disease. We stratified physical examination findings, inflammatory back pain status, and musculoskeletal ultrasonographic findings by arthritis vs no arthritis. We estimated the cumulative incidence of any arthritis and autoimmune disease and calculated corresponding 95% CIs using product-limit (Kaplan-Meier) survival analysis methods to account for censoring. We restricted Kaplan-Meier analyses to any arthritis condition (by ILAR and/or ASAS criteria) and any nonarthritis autoimmune disease, and we excluded other immunologic diseases. We calculated overall and sex-stratified crude incidence rates (IRs) of any arthritis and any autoimmune disease, expressed as the number of cases per 100 000 person-years. We depicted overlap in immune activation signs, arthritis subtypes, and autoimmune diseases in the eFigure 2 in Supplement 1. We completed all analyses using SAS software, version 9.4 (SAS Institute Inc).

## Results

The analyses included 193 patients who met PANS criteria (112 boys [58.0%] and 81 girls [42.0%]; 5 Asian patients [2.6%], 160 White patients [82.9%], 22 Hispanic or Latino patients [11.4%], and 28 multiracial patients [14.5%]) (Table 1).^7,31^ The mean (SD) age at PANS onset was 7.5 (3.5) years. Patients were followed up in the Immune Behavioral Health Clinic for a mean (SD) of 4.0 (2.1) years (from initial clinic encounter to most recent encounter at time of analysis).

**Table 1.** Demographic Characteristics of Consecutive Patients With PANS Seen at a Single Center

Characteristic	Patients (N = 193)
Sex, No. (%)	
Female	81 (42.0)
Male	112 (58.0)
Race, No. (%)	
Asian	5 (2.6)
White	160 (82.9)
Multiracial	28 (14.5)
Hispanic or Latino ethnicity, No. (%)^a^	22 (11.4)
Preexisting neurodevelopmental disorder or neurodivergence, No. (%)^b^	13 (6.7)
Age at first Immune Behavioral Health Clinic visit, mean (SD), y	9.8 (3.9)
Age at most recent Immune Behavioral Health Clinic visit, mean (SD), y	13.9 (4.7)
Immune Behavioral Health Clinic follow-up time, mean (SD), y	4.0 (2.1)
Age at PANS onset, mean (SD), y	7.5 (3.5)
Age at arthritis diagnosis, mean (SD), y (n = 55)	12.7 (3.7)
Age at autoimmune disease diagnosis, mean (SD), y (n = 21)	12.4 (6.4)
Neuropsychiatric symptoms at the initial clinic presentation, No. (%)	
Obsessive-compulsive symptoms	174 (90.2)
Anxiety	172 (89.1)
Irritability, aggression, and/or severely oppositional behaviors	156 (80.8)
Mood dysregulation	153 (79.3)
Sensory dysregulation or amplification^c^	147 (76.2)
Cognitive impairment	145 (75.1)
Sleep disturbances^d^	137 (71.0)
Eating restriction	107 (55.4)
Motor and/or phonic tics	88 (45.6)
Behavioral or developmental regression	86 (44.6)
Urinary symptoms^e^	79 (40.9)
Suicidal ideation or behavior	33 (17.1)

Abbreviation: PANS, pediatric acute-onset neuropsychiatric syndrome.

^a^ Of 22 patients, 19 were White and 3 were multiracial.

^b^ Confirmed autism spectrum disorder (n = 7), suspected autism spectrum disorder (n = 4), other neurodevelopmental disorders (n = 2).

^c^ Sensory dysregulation includes hyperacusis, photophobia, and pain amplification.

^d^ Sleep disturbances include rapid eye movement sleep motor disinhibition, reverse cycling, insomnia, and restless sleep.

^e^ Urinary symptoms include secondary enuresis, involuntary daytime urination, increased urinary frequency, and polyuria.

Proportions of patients with signs of immune activation or inflammation in 3 subcategories are provided in Table 2. Most patients (77.2% [149 of 193]) had at least 1 sign of autoimmunity, immune dysregulation or inflammation, or vasculopathy during a flare. A total of 97 of 179 patients (54.2%) had at least 1 finding of nonspecific autoimmunity, and 69 of 193 patients (35.8%) had at least 1 finding of nonspecific vasculopathy. Fewer patients had a sign of nonspecific immune dysregulation or inflammation (22 of 184 [12.0%]). These proportions were notably higher when estimated among the subset of patients who were tested for all signs in each category (69.2% autoimmunity [27 of 39], 96.3% vasculopathy [26 of 27], and 20.5% immune dysregulation or inflammation [16 of 78]). Approximately one-third of patients with at least 1 sign of autoimmunity, immune dysregulation or inflammation, or vasculopathy showed signs of having markers consistent with at least 2 of these subcategories (eFigure 2A in Supplement 1).

**Table 2.** Laboratory Markers and Physical Signs Of Immune Activation or Inflammation Assessed in a Flare (Neuropsychiatric Symptom Exacerbation)^a^

Marker	No./total No. (%) (N = 193)
**Autoimmune markers (nonspecific) (n = 179)**	
Antinuclear antibody, titer ≥1:80^b^	22/109 (20.2)
Antihistone antibody, high	16/82 (19.5)
Antithyroid antibodies, high^c^	16/114 (14.0)
C1q binding assay, high	27/136 (19.9)
Complement 3, low	12/137 (8.8)
Complement 4, low	44/140 (31.4)
Among those tested for ≥1 marker	
≥1 Positive result	97/179 (54.2)
≥2 Positive results	32/179 (17.9)
Among those tested for all 6 markers	
≥1 Positive result	27/39 (69.2)
≥2 Positive results	14/39 (35.9)
**Immune dysregulation or inflammation markers (nonspecific) (n = 184)**	
Leukopenia, white blood cell count <4000 cells/µL	8/183 (4.4)
Thrombocytosis, platelet count >400 × 10^3^ cells/µL	8/183 (4.4)
C-reactive protein, high	3/118 (2.5)
Erythrocyte sedimentation rate, high	6/96 (6.3)
Among those tested for ≥1 marker	
≥1 Positive result	22/184 (12.0)
≥2 Positive results	2/184 (1.1)
Among those tested for all 4 markers	
≥1 Positive result	16/78 (20.5)
≥2 Positive results	2/78 (2.6)
**Vasculopathy or vascular inflammation markers (nonspecific) (n = 193)**	
von Willebrand factor antigen, high	14/129 (10.9)
d-dimer, high	9/122 (7.4)
Livedo reticularis, present	46/193 (23.8)
Periungual redness, present	21/193 (10.9)
Onychodermal band, abnormally prominent	39/193 (20.2)
Palatal petechiae, present	6/193 (3.1)
Among those tested for ≥1 marker	
≥1 Positive result	69/193 (35.8)
≥2 Positive results	43/193 (22.3)
Among those tested for all 6 markers	
≥1 Positive result	26/27 (96.3)
≥2 Positive results	22/27 (81.5)

SI conversion factors: To convert white blood cells to ×10^9^/L, multiply by 0.001; and platelets to ×10^9^/L, multiply by 1.0.

^a^ All the laboratory and physical examination signs presented were hypothesized to be relevant in the fall of 2012, and the clinicians have attempted to collect the data at the first flare captured in the clinic for all patients. Due to the severe psychiatric symptoms and young age of the study sample, we were not able to complete the standard workup for all patients; denominators are reported for each marker.

^b^ Antinuclear antibody titers: 1:80 (n = 9), 1:160 (n = 8), 1:320 (n = 2), and 1:640 or greater (n = 3).

^c^ Thyroglobulin antibodies and/or thyroperoxidase antibodies.

The cumulative incidence of arthritis by 14 years of age was 28.3% (95% CI, 20.8%-36.3%), and the cumulative incidence of other autoimmune conditions was 7.5% (95% CI, 4.0%-12.4%) (Figure; eTable 1 in Supplement 1). The mean (SD) age was 12.7 (3.7) years for arthritis diagnosis and 12.4 (6.4) years for other autoimmune diagnosis, with only 4 having an autoimmune disorder prior to PANS onset. The IR for developing arthritis (IR, 2120 per 100 000 person-years) was higher than the incidence of other autoimmune disease (IR, 789 per 100 000 person-years), with girls having a higher incidence of other autoimmune disease than boys (IR, 1173 vs 550 per 100 000 person-years) (eTable 2 in Supplement 1).

**Figure.**
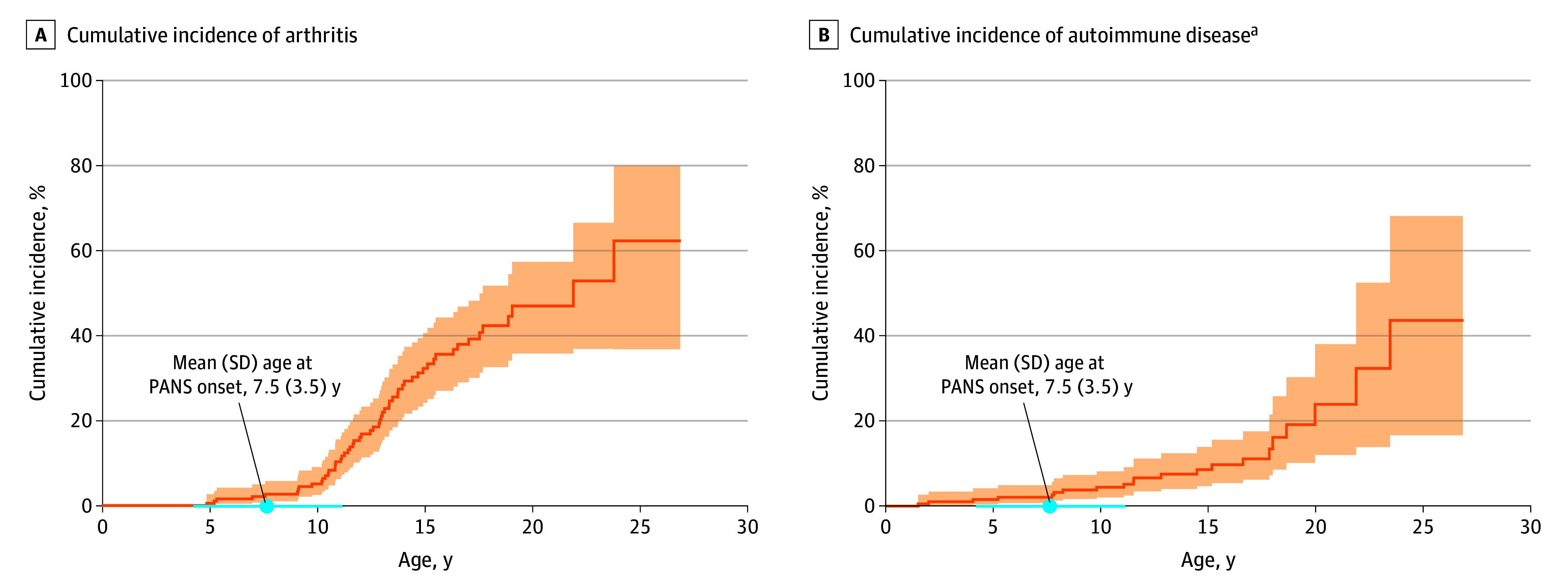
Cumulative Incidence of Arthritis (International League of Associations for Rheumatology and/or Assessment of SpondyloArthritis international Society Criteria) and Autoimmune Disease Among Consecutive Patients With Pediatric Acute-Onset Neuropsychiatric Syndrome (PANS) The shaded areas indicate the 95% CI. ^a^Includes thyroiditis, psoriasis, inflammatory bowel disease, celiac disease, Behcet disease, systemic lupus erythematosus, and diabetes mellitus type I but does not include arthritis.

Among patients who developed arthritis (n = 55), 37 (67.3%) met criteria for ERA, 27 (49.1%) met criteria for spondyloarthritis (SpA) (Table 3), and 15 (27.3%) met criteria for both (eFigure 2B in Supplement 1); 10 patients (18.2%) met the criteria for psoriatic arthritis (PsA). A total of 12 of the 55 patients with arthritis (21.8%) had an additional other autoimmune disease (eFigure 2C in Supplement 1).

**Table 3.** Arthritis Subtypes and Other Autoimmune or Autoinflammatory Diseases Among Consecutive Patients With PANS

Characteristic	Patients (N = 193)
**Arthritis subtypes**	
Any arthritis (meets ILAR and/or ASAS criteria), No. (%)^a^	55 (28.5)
Age at diagnosis of arthritis, mean (SD), y	12.7 (3.7)
Subtype based on ILAR criteria, No. (%)	
Enthesitis-related arthritis	37 (19.2)
Psoriatic arthritis	10 (5.2)
Oligoarticular arthritis	
Persistent	3 (1.6)
Extended	0
RF-positive polyarthritis	0
RF-negative polyarthritis	0
Undifferentiated arthritis	0
Subtype based on ASAS criteria	
Spondyloarthritis, No. (%)	27 (14.0)
Peripheral, No./total No.	26/27^b^
Axial, No./total No.	7/27^b^
**Autoimmune or inflammatory diseases (beyond arthritis)**	
Any autoimmune disease, No. (%)^a^^,^^c^	21 (10.9)
Age at diagnosis, mean (SD), y	12.4 (6.4)
Thyroiditis	8 (4.1)
Psoriasis	5 (2.6)
Inflammatory bowel disease^d^	5 (2.6)
Celiac disease	4 (2.1)
Behcet disease	2 (1.0)
Systemic lupus erythematosus	1 (0.5)
Type 1 diabetes	1 (0.5)
≥1 Comorbid autoimmune disease (beyond arthritis), No./total No. (%)	5/21 (23.8)
Other immunologic diseases, No. (%)	
Primary immunodeficiency	8 (4.1)
Chronic urticaria	4 (2.1)
Periodic fever, aphthous stomatitis, pharyngitis, adenitis	2 (1.0)
Eosinophilic esophagitis	2 (1.0)

Abbreviations: ASAS, Assessment of SpondyloArthritis international Society; ILAR, International League of Associations for Rheumatology; PANS, pediatric acute-onset neuropsychiatric syndrome; RF, rheumatoid factor.

^a^ See eFigure 2 in Supplement 1 for details on overlapping arthritis and autoimmune diagnoses.

^b^ Percentages not given because some patients fulfilled criteria for both peripheral and axial spondyloarthritis.

^c^ Criteria for classifying patients with these autoimmune conditions are listed in eAppendix 2 in Supplement 1.

^d^ Including Crohn disease (n = 2), ulcerative colitis (n = 1), and related to common variable immunodeficiency (n = 2).

Joint examinations of the 55 patients with arthritis revealed tenderness in the distal interphalangeal (DIP) joints of 45 children (81.8%) and spinous process of 44 children (80.0%); 34 of the 138 children (24.6%) without arthritis also had tenderness of the spinous process (Table 4). Among those with arthritis who underwent joint ultrasonography (n = 40), the most common findings were effusions in 31 patients (77.5%), capsular thickening (capsulitis) in 22 patients (55.0%), and synovitis in 22 patients (55.0%); there were no cases of capsulitis in the 27 patients without arthritis who underwent joint ultrasonography. Nail pitting was present in 9 of the 55 patients with arthritis (16.4%) and 13 of the 138 patients without arthritis (9.4%). Psoriasis was seen in 5 of the 193 patients in the total cohort (2.6%) (Table 3).

**Table 4.** Musculoskeletal and Nail Characteristics Among Consecutive Patients With PANS

Characteristic	Met arthritis criteria	Did not meet arthritis criteria
**Musculoskeletal and nail characteristics, stratified by arthritis status**		
No.	55	138
Inflammatory back pain		
Meets Calin criteria for inflammatory back pain, No. (%)	35 (63.6)	17 (12.3)
Physical examination (n = 193)		
Prevalence of tenderness, No. (%)		
Distal interphalangeal joints	45 (81.8)	39 (28.3)
Spinous process tenderness	44 (80.0)	34 (24.6)
Sacroiliacjoint tenderness	38 (69.1)	30 (21.7)
Achilles tendon insertion (ie, heel enthesitis)	31 (56.4)	19 (13.8)
Other characteristics		
Nail pitting, No. (%)	9 (16.4)	13 (9.4)
**Joint ultrasonography (n = 67)**		
No.	40	27
Normal, No. (%)	3 (7.5)	16 (59.3)
Abnormal, No. (%)^a^	37 (92.5)	11 (40.7)
Joint effusion	31	8^b^
Capsular thickening (capsulitis)	22	0
Synovial thickening or proliferation (synovitis)	22	5^b^
Peritendinous or entheseal effusion	2	1
Tendinous thickening or tenosynovitis	1	0
Bone erosion	1	0
Ganglion cyst	1	0
Bursitis	0	0
Bone edema	0	0
Joint narrowing	0	0
Joint widening	0	0
Subchondral sclerosis	0	0
Other^c^	4	1

Abbreviation: PANS, pediatric acute-onset neuropsychiatric syndrome.

^a^ Percentages of individual abnormal findings not given because some patients had more than one of these findings.

^b^ Although typically a primary indicator of arthritis, at the time of data collection, the synovitis and effusions detected on ultrasonograms were subtle (for these 9 patients); to be conservative with our classification, we decided not to classify these patients as meeting criteria for arthritis, but 5 patients progressed with the arthritis and now meet criteria (this change was not included in the cumulative incidence analysis; Figure). Numbers do not add up due to overlap in ultrasonographic findings.

^c^ Includes hypoechogenic nodule, suprapatellar echogenic fat vs artifact, cysts, or small fibrotic nodules.

## Discussion

To our knowledge, this is the largest report of patients with PANS followed up longitudinally for inflammatory signs and diseases. A previous study reported that among the first 47 patients who presented to the clinic, one-third developed arthritis, but the study did not subtype the arthritis or describe autoimmune conditions.^2^ Thus, we pursued this study to give greater detail on a larger patient population. We observed an unexpectedly high frequency of immune activation signs, arthritis, and autoimmune disease, suggesting that PANS itself is part of an inflammatory diathesis, akin to neuropsychiatric lupus or Sydenham chorea, where neuroinflammation is comorbid with arthritis and other inflammatory signs.^37,38,39^

Several abnormalities (livedo reticularis; periungual redness and swelling; and prominent onychodermal bands, akin to Terry nails, a known nonspecific indicator of systemic inflammation^40^) may represent a low-level small vessel vasculitis in PANS, consistent with our findings of elevated level of von Willebrand factor antigen and d-dimer in a subset of patients.^41,42^ Most of the patients did not have an active infection at the time of presentation; thus, we do not suspect that high d-dimer represents a sign of overt infection. We do not yet know how laboratory variables cluster together or cluster with physical examination findings of vasculopathy and arthritis.

The high level of antihistone antibodies are another remarkable finding. We proactively evaluated this autoantibody given concern for drug-induced lupus. In addition, a small study indicated that antihistone antibodies were associated with neuropsychiatric lupus.^43^ Although antihistone antibodies were noted in our PANS cohort, they were not elevated in the Karolinska PANS cohort.^44^ We plan to study high antihistone antibodies in this population and compare with healthy controls.

Inflammation in this cohort does not provoke a significant acute phase response because PANS-related inflammation is likely associated with ERA and juvenile SpA. There have been 4 proposed classification criteria for juvenile SpA, and none of them included C-reactive protein and erythrocyte sedimentation rate.^45^ The adult forms of these diseases may include erythrocyte sedimentation rate elevation likely due to more advanced inflammation, unlike in our patient population, whom we are screening and diagnosing arthritis at an early stage. In addition, early presentation of axial SpA is not associated with elevated C-reactive protein and erythrocyte sedimentation rate.^46^

We report the novel finding of capsulitis in 22 of 40 patients (55.0%) with arthritis but no capsulitis in the 27 patients who underwent joint ultrasonography and did not meet criteria for arthritis. To our knowledge, this is the first report of capsulitis in children with peripheral arthritis, with the exception of pediatric temporomandibular joint arthritis.^47^ Capsulitis and DIP joint tenderness are reported in adults with PsA.^48,49^ Given the high rate of nail pitting and enthesitis in patients with capsulitis and DIP joint tenderness, it is possible that capsulitis and DIP joint tenderness may be early markers of PsA or associated with PsA among patients with PANS.

Arthritis-type descriptions in this particular cohort are detailed in a case series of 7 patients in which the figures indicate a distinction between the synovium and the capsule.^50^ Previous ultrasonography studies of juvenile arthritis were based on old technology and may not have been able to distinguish between synovitis and capsulitis. Therefore, the finding of capsulitis in our cohort may not be novel but based on newer ultrasonographic technology (eAppendix 3 in Supplement 1).

The incidence of DIP joint tenderness (81.8%) is much higher than in typical juvenile arthritis cohorts. Hemke et al^51^ found that 4.4% of patients with oligoarticular and polyarticular juvenile arthritis had DIP joint involvement at initial presentation. Considering that pain amplification has also been observed in our PANS cohort and reported in PsA (range, 10%-27%) and axial SpA (range, 4%-25%),^52^ this finding brings up the possibility that DIP joint tenderness could represent an enhanced pain response. However, given the high prevalence of nail pitting, often associated with enthesitis and/or arthritis at the DIP joint (among patients with PsA), we suspect the finding of DIP joint tenderness could be an early sign that these patients have a condition associated with PsA.

Spinous process tenderness was the most common enthesitis point, seen in 80.0% of the patients with arthritis and 24.6% of the patients without arthritis. There has not been prior research on this finding in children, to our knowledge. For this study, we were strict with our arthritis classification, and we may be underreporting it because almost one-fourth of the children who did not have a diagnosis of arthritis had spinous process tenderness and/or Achilles tendon insertion tenderness.

For many patients with PANS, findings of arthritis on physical examination are subtle, even without immunomodulation. This phenomenon is well described in PsA, where the arthritis is often “dry” (without effusion or swelling) and insidious, and for acute rheumatic fever, where the arthritis pain is often incommensurate to examination findings. Musculoskeletal ultrasonography has become more accessible and is instrumental in the diagnosis of dry arthritis. Therefore, we have a low threshold for ordering imaging. Earlier diagnosis enables earlier treatment for arthritis among children suffering from a complex condition such as PANS.

Physical examinations may reveal early signs of arthritis, such as nail pitting, often a factor associated with arthritis.^53^ Joint warmth and abnormalities on imaging are useful objective signs of joint inflammation. We recently reexamined clinic records for 9 of the patients with subtle abnormalities on musculoskeletal ultrasonography (Table 4) that did not meet ILAR or ASAS criteria for arthritis and 5 of those patients now meet criteria for arthritis.

Although nail pitting is very common in this patient population (9.4%-16.4%), the diagnosis of psoriasis is less common (2.6%), likely reflecting our rigorous classification of autoimmune diseases (eAppendix 2 in Supplement 1). Although we often find subtle evidence of psoriasis on examination, we do not always refer the patient to dermatology for a confirmatory diagnosis due to patient impairment and caregiver burden.^1^

Most of the patients had an overlapping or interconnected subgroup of diagnoses (ERA, PsA, and SpA), which is distinctly different when compared with rheumatoid arthritis and other forms of juvenile arthritis. This subgroup is overrepresented, while other subtypes of juvenile idiopathic arthritis (JIA) are not represented (oligoarticular-extended arthritis, rheumatoid factor–negative or rheumatoid factor–positive polyarticular arthritis, systemic arthritis), suggesting a pathophysiological link between this subgroup and PANS.

Enthesitis-related arthritis is part of ILAR classification criteria for JIA and not applied to adults. However, ERA is thought to be a forme fruste of PsA and SpA. To our knowledge, there are not yet validated criteria for juvenile SpA, and many pediatric patients with inflammatory back pain are classified as having ERA given that they do not meet ASAS criteria for SpA (because criteria were developed using primarily adult data). Both PsA and SpA are insidious in their presentation, and separating the ERA and SpA diagnoses is an artificial construct in youth; hence, we applied the ERA classification criteria despite the patients being 16 years of age or older.

IL-17 has been implicated in (1) animal models of PANDAS, (2) subtypes of arthritis in our PANS or PANDAS cohort, (3) pediatric OCD, and (4) psoriasis.^26,54^ In murine models, repeated GAS infection has been shown to induce a robust Th17 response in nasal-associated lymphoid tissue.^26^ This model showed that GAS exposure promoted migration and persistence of GAS-specific Th17 cells to the brain, leading to neurovascular dysfunction. Moreover, GAS-specific Th17 cells are found in the tonsils of patients exposed to GAS.^26,54^ Interleukin-17 is likely a factor associated with the 3 overlapping arthritis types that we see in our cohort^55,56^ and has also been reported to be present in high levels in pediatric patients with OCD.^57^ Similar to the PANDAS mouse model, repeated GAS infections may also play a role in psoriasis, thus making the nail pitting finding in our cohort relevant because this is a common finding in psoriasis.^58^

There is a high rate of psychiatric symptoms (including OCD and depression) in psoriasis.^59^ Adults with SpA have a high rate of OCD and anger-hostility compared with controls and have a high rate of depression, anxiety, and fatigue.^60^ In a pediatric study, McHugh et al^61^ noted that patients with arthritis (ERA, SpA, and polyarticular arthritis) who had more active arthritis had worse psychological functioning and more emotional disturbances.

The proportion of arthritis and autoimmune disease in our cohort is significantly higher than observed in the general population, where the prevalence of JIA is estimated to be 20.5 cases per 100 000 people and the annual incidence is estimated to be 7.8 cases per 100 000.^62^ In contrast, we observed a cumulative incidence of 28.3% by 14 years of age and incidence rate of 2120 per 100 000 people per year for juvenile-onset arthritis (ILAR and/or ASAS criteria). Although ASAS criteria (for SpA) and Calin criteria (for inflammatory back pain) have not been formally validated and are thought to have poor sensitivity in children, many of the patients fit these criteria.

The prevalence of autoimmune disease (including rheumatoid arthritis) is estimated to be 4.5% to 9.4% of the general adult population, and the typical age of diagnosis is in adulthood.^63,64,65^ The prevalence of autoimmune disease in children is much lower than in the general population, as only a handful of autoimmune diseases have juvenile onset.^66^ However, within our cohort, the cumulative incidence of other autoimmune disease (not including arthritis) by 14 years of age was 7.5%. A previously published prevalence study of “autoimmune phenomenon” in PANDAS (based on autoantibodies to thyroid and celiac disease) did not formally evaluate ERA, PsA, and SpA, as these diagnoses are based on physical examination and not autoantibodies.^67^

We plan to study how inflammatory variables cluster in our cohort and how laboratory markers correlate with physical examination findings of vasculopathy at presentation and are associated with arthritis and psychiatric symptoms trajectories. Pediatric reference ranges have not been rigorously established for certain laboratory results, such as antihistone antibodies and von Willebrand factor antigen. Thus, we aim to compare all the markers established in this manuscript to an age- and sex-matched control population.

Our data collection did not include reviewing the electronic medical record for all tender joints. Instead, we focused our review on clinical features that distinguished this patient population from typical cohorts with JIA (ie, DIP joint tenderness, spinous process tenderness). We plan on reporting the full 66 swollen and 68 tender joint count, and the Mander enthesitis index at clinical presentation and the evolution of the arthritis in this cohort. We plan to do a cluster analysis and predictor analysis to understand the findings of DIP joint tenderness and nail pitting. This analysis will include controlling for confounding variables including pain amplification (using the American College of Rheumatology fibromyalgia tool measured at every visit for the past decade).^68^

### Limitations

This study has some limitations. We did not have a control sample to make a direct comparison, but this is a planned future project dependent on funding. We did not study medications that might be associated with risk of arthritis, but this will be a future study. Our study is limited by missing data. Changes in our clinical standards and the complexity of the childrens’ psychiatric presentation challenged the completion of comprehensive testing and examinations. We initially focused on infectious triggers of PANS or PANDAS and performed rheumatologic examinations only for children with joint pain. As we learned that children were underreporting joint symptoms, we standardized our evaluations to include a comprehensive rheumatologic evaluation. Laboratory data were missed for children with needle phobia, small blood volume due to young age, challenging behavior, and/or lack of insurance. Thus, our results are likely more accurate for recent and older patients and those with less-severe psychiatric illness. Although our findings may be relevant to other postinfectious neuropsychiatric conditions, the results should not be generalized beyond patients with PANS.

## Conclusions

Pediatric acute-onset neuropsychiatric syndrome is currently characterized as an abrupt-onset psychiatric illness likely involving the basal ganglia. Our findings demonstrate that a significant proportion of patients with PANS have evidence of subtle systemic inflammation and raise the possibility that the psychiatric symptoms reflect a brain response to a global process.

The patients had elevated markers suggesting nonspecific autoimmunity or inflammation and small vessel vascular involvement at psychiatric illness presentation and a heightened risk for developing arthritis and/or other autoimmune or inflammatory diseases compared with the general pediatric population. Low C4 gene copy has been previously described as a risk factor for developing arthritis in our cohort.^69^ Although the laboratory findings of inflammation are subtle, the inflammation (typically a microscopic finding) is significant enough to be detected on joint ultrasonograms. Addressing inflammation may be critical in preventing the development of arthritis and other autoimmune diseases, but clinical trials are needed to determine the association of anti-inflammatories with psychiatric symptoms in this patient population.

## Notes

Supplement 1. **eAppendix 1.** Pediatric Autoimmune Neuropsychiatric Disorder Associated With Streptococcal Infection (PANDAS) and Pediatric Acute-Onset Neuropsychiatric Syndrome (PANS) Criteria
eMethods.
**eFigure 1.** 22-Year-Old Patient With PANS (Relapsing-Remitting Course) **eAppendix 2.** Classification Criteria **eAppendix 3.** Specifications of Musculoskeletal US Technology Used in This Study **eFigure 2.** Overlap Between Available Evidence of (a) Laboratory and Physical Signs of Immune Activation in Flare and (b) Arthritis Subtypes and (c) Arthritis Subtypes and Other Autoimmune Diseases Among Consecutive Patients With PANS **eTable 1.** Cumulative Incidence of Arthritis and Autoimmune Disease for Different Cumulative Time Frames by Age (Years) Among Consecutive Patients With PANS, N=193 **eTable 2.** Incidence Rates for Arthritis and Autoimmune Disease Among Consecutive Patients With PANS, N=193

Supplement 2.
Data Sharing Statement
