# Supplementary material for: Development of Autoimmune Diseases Among Children With Pediatric Acute-Onset Neuropsychiatric Syndrome
Source: JAMA Netw Open. 2024 Jul 30;7(7):e2421688. doi: 10.1001/jamanetworkopen.2024.21688 (PMC11289697; doi:10.1001/jamanetworkopen.2024.21688)
Supplement: Supplement 1. — eAppendix 1. Pediatric Autoimmune Neuropsychiatric Disorder Associated With Streptococcal Infection (PANDAS) and Pediatric Acute-Onset Neuropsychiatric Syndrome (PANS) Criteria eMethods. eFigure 1. 22-Year-Old Patient With PANS (Relapsing-Remitting Course) eAppendix 2. Classification Criteria eAppendix 3. Specifications of Musculoskeletal US Technology Used in This Study eFigure 2. Overlap Between Available Evidence of (a) Laboratory and Physical Signs of Immune Activation in Flare and (b) Arthritis Subtypes and (c) Arthritis Subtypes and Other Autoimmune Diseases Among Consecutive Patients With PANS eTable 1. Cumulative Incidence of Arthritis and Autoimmune Disease for Different Cumulative Time Frames by Age (Years) Among Consecutive Patients With PANS, N=193 eTable 2. Incidence Rates for Arthritis and Autoimmune Disease Among Consecutive Patients With PANS, N=193 [file jamanetwopen-e2421688-s001.pdf]

# Supplementary Online Content

Ma M, Masterson EE, Gao J, et al. Development of autoimmune diseases among children with pediatric acute-onset neuropsychiatric syndrome. *JAMA Netw Open.* 2024;7(7):e2421688. doi:10.1001/jamanetworkopen.2024.21688

**eAppendix 1.** Pediatric Autoimmune Neuropsychiatric Disorder Associated With Streptococcal Infection (PANDAS) and Pediatric Acute-Onset Neuropsychiatric Syndrome (PANS) Criteria
**eMethods.**
**eFigure 1.** 22-Year-Old Patient With PANS (Relapsing-Remitting Course)
**eAppendix 2.** Classification Criteria
**eAppendix 3.** Specifications of Musculoskeletal US Technology Used in This Study
**eFigure 2.** Overlap Between Available Evidence of (a) Laboratory and Physical Signs of Immune Activation in Flare and (b) Arthritis Subtypes and (c) Arthritis Subtypes and Other Autoimmune Diseases Among Consecutive Patients With PANS
**eTable 1.** Cumulative Incidence of Arthritis and Autoimmune Disease for Different Cumulative Time Frames by Age (Years) Among Consecutive Patients With PANS, N=193
**eTable 2.** Incidence Rates for Arthritis and Autoimmune Disease Among Consecutive Patients With PANS, N=193

This supplementary material has been provided by the authors to give readers additional information about their work.

© 2024 Ma M et al. *JAMA Network Open.*

**eAppendix 1.** Pediatric Autoimmune Neuropsychiatric Disorder Associated With Streptococcal Infection (PANDAS) and Pediatric Acute-Onset Neuropsychiatric Syndrome (PANS) Criteria

| PANDAS Criteria<br>1998 |
|---|
| 1. Presence of diagnosis of OCD and/or tic disorder |
| 2. Pediatric onset |
| 3. Episodic course |
| 4. Association with group A beta-hemolytic streptococcus infection |
| 5. Association with neurologic abnormalities |

| PANS Criteria<br>2012 |
|---|
| 1. Abrupt, dramatic onset of OCD or severely restricted food intake |
| 2. Concurrent presence of additional neuropsychiatric symptoms, (with similarly severe and acute onset), from at least two of the following seven categories<br>A. Anxiety<br>B. Emotional lability and/or depression<br>C. Irritability, aggression, and/or severely oppositional behaviors<br>D. Behavioral (developmental) regression<br>E. Deterioration in school performance<br>F. Sensory or motor difficulties<br>G. Somatic signs or symptoms, including sleep disturbances, enuresis, or urinary frequency |
| 3. Symptoms are not better explained by a known neurologic or medical disorder, such as Sydenham chorea, systemic lupus erythematosus, Tourette syndrome, or others |

Swedo SE, Leonard HL, Garvey M, et al. Pediatric autoimmune neuropsychiatric disorders associated with streptococcal infections: clinical description of the first 50 cases. Am J Psychiatry. 1998 Feb;155(2):264-71.
Swedo S, Leckman J, Rose N: From research subgroup to clinical syndrome: Modifying the PANDAS criteria to describe PANS (pediatric acute-onset neuropsychiatric syndrome). Pediatr Therapeutics 2:1–8, 2012

© 2024 Ma M et al. *JAMA Network Open.*

**eMethods. Inclusion/exclusion flowchart in patients with Pediatric Acute-onset Neuropsychiatric Syndrome (PANS).**
Inclusion/exclusion criteria for this study. Before entering our PANS clinic, patients are prescreened for the likelihood of having PANS using the following: family patient questionnaire, review of outside records and/or discussion with primary medical doctor. Patients must have had an established primary medical doctor for more than 3 years to gain entry into the PANS clinic.

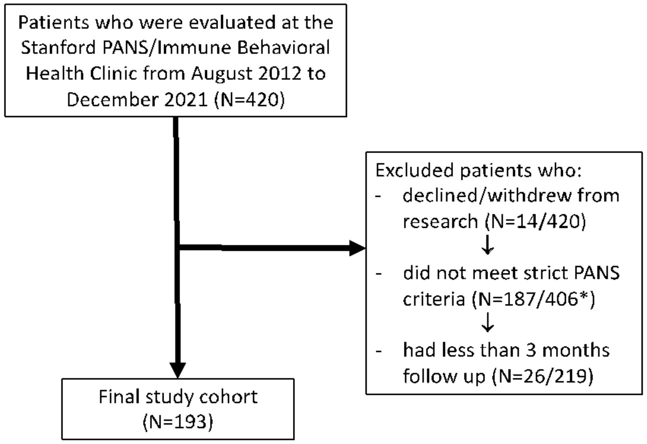


*Patients who did not consent to research did not have their charts reviewed for evaluation of meeting PANS criteria or follow up time. Only the 406 patients consenting to research had charts reviewed for meeting PANS criteria and follow up time.

© 2024 Ma M et al. *JAMA Network Open.*

**eFigure 1.** 22-Year-Old Patient With PANS (Relapsing-Remitting Course). Relapses have been associated with streptococcal infections and had eventual development of **a.** periungual redness and swelling involving all fingers and **b.** prominent onychodermal band involving all fingers. This patient also had eventual diagnosis of arthritis involving the wrists, fingers, and toes.

a.

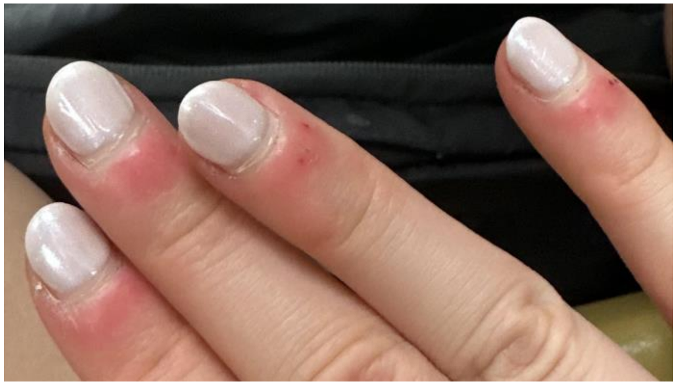

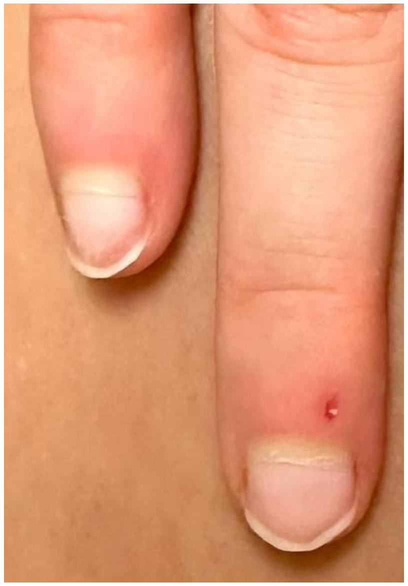

b.

© 2024 Ma M et al. *JAMA Network Open.*

**eAppendix 2.** Classification Criteria

a. **Classification criteria for arthritis**

| | **Definition/Criteria** |
|---|---|
| International League of Associations of Rheumatology (ILAR)[a] | |
| Enthesitis-Related Arthritis (ERA) | Arthritis and enthesitis or arthritis or enthesitis with at least 2 of the following:<br>. Sacroiliac joint tenderness or inflammatory back pain<br>. HLA-B27 antigen<br>. Onset of arthritis in a male over 6 years of age<br>. Acute anterior uveitis<br>. History of ankylosing spondylitis, enthesitis related arthritis, sacroiliitis with inflammatory bowel disease, or acute anterior uveitis in a first-degree relative |
| Juvenile Psoriatic Arthritis (PsA) | Arthritis and psoriasis or arthritis and at least 2 of the following:<br>. Dactylitis<br>. Nail-pitting or onycholysis<br>. Psoriasis in a first-degree relative |
| Oligoarticular Juvenile Idiopathic Arthritis (persistent and extended) | Arthritis affecting 1 to 4 joints during the first 6 months of disease<br>Persistent: no more than 4 joints affected throughout disease course<br>Extended: more than 4 joints affected after the first 6 months of disease |
| RF positive polyarthritis | Arthritis affecting >5 joints, positive rheumatoid factor at least 3 months apart during first 6 months of disease |
| RF negative polyarthritis | Arthritis affecting >5 joints, negative rheumatoid factor |
| Undifferentiated Arthritis | Arthritis that fulfills no category or in >2 of above |
| Spondyloarthritis (Axial[b] and Peripheral[c]) | **Axial Spondyloarthritis ASAS criteria**<br>1. Back pain equal to and more than 3 months, and<br>2. Age at onset < 45 years, and<br>3. Sacroiliitis on imaging (active acute inflammation on MRI highly suggestive of sacroiliitis associated with spondyloarthritis, or definite radiographic sacroiliitis), plus at least 1 of the following features:<br>(A) inflammatory back pain<br>(B) arthritis<br>(C) enthesitis (heel)<br>(D) uveitis<br>(E) dactylitis |

© 2024 Ma M et al. *JAMA Network Open.*

| | |
|---|---|
| | (F) psoriasis<br>(G) Crohn’s disease/ulcerative colitis<br>(H) good response to NSAIDs<br>(I) family history of spondyloarthritis<br>(J) HLA-B27<br>(K) elevated CRP<br>or<br>4. HLA-B27 plus at least 2 of the following features:<br>(A) inflammatory back pain<br>(B) arthritis<br>(C) enthesitis (heel)<br>(D) uveitis<br>(E) dactylitis<br>(F) psoriasis<br>(G) Crohn’s disease/ulcerative colitis<br>(H) good response to NSAIDs<br>(I) Family history of spondyloarthritis<br>(J) HLA-B27<br>(K) Elevated CRP<br>**Peripheral Spondyloarthritis ASAS Criteria**<br>1. Arthritis, or enthesitis, or dactylitis, and<br>2. At least one of the following features:<br>(A) psoriasis<br>(B) inflammatory bowel disease<br>(C) preceding infection<br>(D) HLA-B27<br>(E) uveitis<br>(F) sacroiliitis on imaging (radiographs or MRI)<br>or<br>3, At least two of the following features:<br>(A) arthritis<br>(B) enthesitis<br>(C) dactylitis<br>(D) inflammatory back pain in the past<br>(E) positive family history of spondyloarthritis |

[a]Petty RE, et al. ILAR classification of juvenile idiopathic arthritis: Second revision, Edmonton 2001. J Rheumatol 2004; 31(2): 390-2.
[b]Rudwaleit M, Landewé R, van der Heijde D, Listing J, Brandt J, et al. The development of Assessment of SpondyloArthritis international Society classification criteria for axial spondyloarthritis (part I): classification of paper patients by expert opinion including uncertainty appraisal. Ann Rheum Dis. 2009 Jun;68(6):770-6.
[c] Rudwaleit M, van der Heijde D, Landewé R, Akkoc N, Brandt J, et al. The Assessment of SpondyloArthritis International Society classification criteria for peripheral spondyloarthritis and for spondyloarthritis in general. Ann Rheum Dis. 2011 Jan;70(1):25-31.

© 2024 Ma M et al. *JAMA Network Open.*

**b. Classification criteria for autoimmune disease and other inflammatory conditions diagnosed by rheumatologists within the Immune Behavioral Health Clinic**

| | |
|---|---|
| Behcet's disease[a] | . Recurrent oral ulcers (at least 3 times in 12 months)<br>Plus ≥ 2 of the following:<br>. Recurrent genital ulceration<br>. Eye lesions<br>. Skin lesions<br>. Pathergy test |
| Systemic Lupus Erythematosus (SLE)[b,c] | **American College Rheumatology (4 of 11)**<br>. Malar rash<br>. Photosensitivity<br>. Discoid rash<br>. Oral ulcers<br>. Arthritis<br>. Serositis<br>. Renal disorder<br>. Neurologic disorder<br>. Hematologic disorder<br>. ANA<br>. Immunologic disorder<br>**Systemic Lupus International Collaborating Clinics (4 of 17 or biopsy proven lupus nephritis)**<br>. Acute cutaneous lupus<br>. Chronic cutaneous lupus<br>. Alopecia<br>. Oral or nasal ulcers<br>. Joint disease<br>. Serositis<br>. Renal disorder<br>. Neurologic disorder<br>. Hematologic disorder (hemolytic anemia, leukopenia or lymphopenia, thrombocytopenia)<br>. ANA<br>. Anti-dsDNA, Anti-Sm, Antiphospholipid antibody<br>. Low complement<br>. Direct Coombs |

© 2024 Ma M et al. *JAMA Network Open.*

| | |
|---|---|
| Periodic fever, aphthous stomatitis, pharyngitis, adenitis (PFAPA)[d] | Diagnosed by a pediatric infectious disease and/or pediatric rheumatologist at an academic center with clinical features such as episodes of high fevers that occur with regularity every 3-6 weeks, begin in early childhood. Fever episodes generally lasting up to 3-6 days, small non-scarring aphthous ulcers, non-exudative pharyngitis, and/or cervical adenitis. Asymptomatic between flares with normal growth. |

[a]Criteria for diagnosis of Behçet's disease. International Study Group for Behçet's Disease. Lancet. 1990 May 5;335(8697):1078-80.
[b]Petri M, Orbai AM, Alarcon GS, Gordon C, Merrill JT, Fortin PR, et al. Derivation and validation of the Systemic Lupus International Collaborating Clinics classification criteria for systemic lupus erythematosus. Arthritis Rheum 2012;64:2677-86.
[c]Hochberg MC. Updating the American College of Rheumatology revised criteria for the classification of systemic lupus erythematosus. Arthritis Rheum. 1997 Sep;40(9):1725.
[d]Marshall GS, Edwards KM, Lawton AR. PFAPA syndrome. Pediatr Infect Dis J. 1989 Sep;8(9):658-9.

**c. Classification criteria for autoimmune disease and other inflammatory conditions diagnosed by board-certified sub-specialists outside of the Immune Behavioral Health Clinic**

| | |
|---|---|
| Celiac Disease | Diagnosed by a pediatric gastroenterologist. Must have either biopsy proven celiac disease or high risk allele (HLA-DQ2 or HLA-DQ8) coupled with high tissue transglutaminase antibodies. |
| Type I Diabetes Mellitus | Diagnosed by a pediatric endocrinologist with elevated A1C, fasting plasma glucose, abnormal oral glucose tolerance test, or classic symptoms of hyperglycemia with requirement of insulin. |
| Chronic Urticaria | Documented by a dermatologist with multi-week corticosteroid treatment; chronic defined as six or more weeks. |
| Eosinophilic Esophagitis | Diagnosed by all of the following:<br>1. symptoms of esophageal dysfunction, or concomitant atopic conditions, or endoscopic findings of rings, furrows, exudates, edema, stricture, narrowing, and crepe-paper mucosa, and<br>2. ≥15 eos/hpf (~60 eos/mm2) on esophageal biopsy, and<br>3. Exclusion of non-EoE disorders that cause or potentially contribute to esophageal eosinophilia. |
| Thyroiditis | History of autoimmune thyroiditis: Patient had to meet one of the following: (1) an ultrasound showing thyroid fullness; (2) elevated thyroid antibodies (TPO or anti-thyroglobulin) on two separate occasions; (3) diagnosed by pediatric endocrinologist. |
| Psoriasis | Diagnosed by a dermatologist. |
| Inflammatory Bowel Disease (IBD) | Documented clinical diagnosis from a pediatric gastroenterologist. |

© 2024 Ma M et al. *JAMA Network Open.*

**eAppendix 3.** Specifications of Musculoskeletal US Technology Used in This Study

All musculoskeletal US examinations were performed either on a GE LOGIQ E9 machine (GE Healthcare Inc., Princeton, NJ) utilizing a 6-15 MHz linear transducer, or a Siemens ACUSON Sequoia machine (Siemens Healthineers AG, Erlangen, Germany) utilizing an 18 MHz linear transducer. Settings on the machine were optimized for superficial musculoskeletal examinations. Both grayscale and color Doppler images of the target joints were acquired and saved on the institute's Picture Archiving and Communication System (PACS). For the fingers and toes, dedicated still images of the metacarpophalangeal and metatarsophalangeal joints were saved, while the proximal and distal interphalangeal joints were scanned and saved together.

© 2024 Ma M et al. *JAMA Network Open.*

**eFigure 2.** Overlap Between Available Evidence of (a) Laboratory and Physical Signs of Immune Activation in Flare and (b) Arthritis Subtypes and (c) Arthritis Subtypes and Other Autoimmune Diseases Among Consecutive Patients With PANS

<table>
<tr>
<th>A. Overlap of autoimmune [a], immune dysregulation [b], and vasculopathy markers [c] among 149 patients with at least one positive marker</th>
<th>B. Overlap of arthritis subtypes among 55 patients diagnosed with arthritis</th>
<th>C. Overlap of arthritis subtypes and other autoimmune diseases among 64 patients diagnosed with arthritis and/or autoimmune diseases</th>
</tr>
<tr>
<td>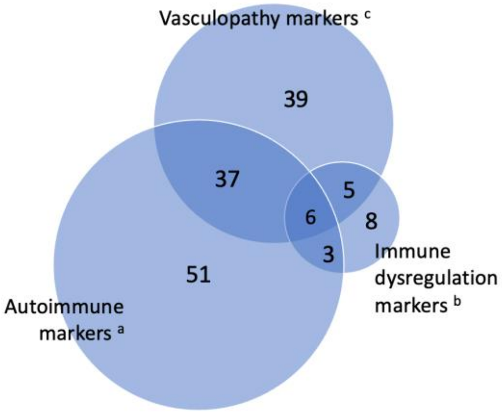
</td>
<td>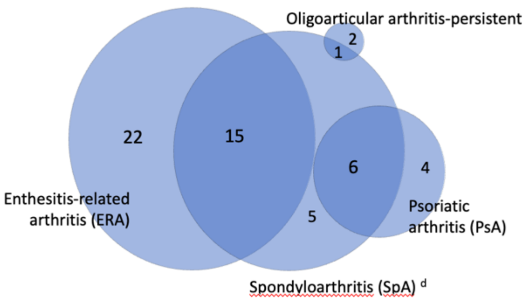
</td>
<td>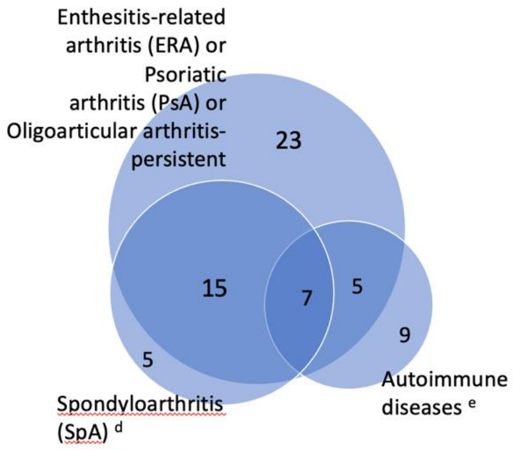
</td>
</tr>
</table>

[a]Positive antinuclear antibody (ANA), high anti-histone antibody, high anti-thyroglobulin antibody, elevated C1q binding assay, low Complement 3 (C3), low Complement 4 (C4)
[b]Leukopenia, thrombocytosis, elevated C-reactive protein, elevated erythrocyte sedimentation rate
[c]Livedo reticularis, onychodermal band, periungual redness, palatal petechiae, elevated von Willebrand factor antigen, elevated D-dimer
[d]Includes peripheral and axial subtypes
[e]Thyroiditis, Psoriasis, Inflammatory bowel disease, Celiac disease, Behcet's disease, Systemic lupus erythematosus, Diabetes mellitus Type I

© 2024 Ma M et al. *JAMA Network Open.*

**eTable 1.** Cumulative Incidence of Arthritis and Autoimmune Disease[b] for Different Cumulative Time Frames by Age (Years) Among Consecutive Patients With PANS, N=193[c]

| **Cumulative time frame** | **juvenile-onset arthritis (ILAR and/or ASAS criteria)** | | | | **other autoimmune diseases[b]** | | | |
|---|---|---|---|---|---|---|---|---|
| **At age (years)** | **Number at risk** | **Number censored** | **Number Events** | **Cumulative Risk, % (95% CI)[a]** | **Number at risk** | **Number censored** | **Number Events** | **Cumulative Risk, % (95% CI)[a]** |
| | | | | | | | | |
| **1** | 193 | 0 | 0 | 0 | 193 | 0 | 2 | 0 |
| **2** | 193 | 0 | 0 | 0 | 191 | 0 | 0 | 1.0 (0.2, 3.4) |
| **3** | 193 | 1 | 0 | 0 | 191 | 1 | 0 | 1.0 (0.2, 3.4) |
| **4** | 192 | 1 | 1 | 0 | 190 | 1 | 1 | 1.0 (0.2, 3.4) |
| **5** | 190 | 1 | 2 | 0.5 (0.05, 2.7) | 188 | 1 | 1 | 1.6 (0.4, 4.2) |
| **6** | 187 | 6 | 1 | 1.6 (0.4, 4.2) | 186 | 6 | 0 | 2.1 (0.7, 4.9) |
| **7** | 180 | 8 | 1 | 2.1 (0.7, 5.0) | 180 | 9 | 2 | 2.1 (0.7, 4.9) |
| **8** | 171 | 8 | 0 | 2.7 (1.0, 5.8) | 169 | 8 | 1 | 3.2 (1.3, 6.5) |
| **9** | 163 | 10 | 4 | 2.7 (1.0, 5.8) | 160 | 11 | 1 | 3.8 (1.7, 7.3) |
| **10** | 149 | 9 | 8 | 5.1 (2.5, 9.0) | 148 | 11 | 0 | 4.4 (2.1, 8.2) |
| **11** | 132 | 16 | 8 | 10.3 (6.2, 15.6) | 137 | 19 | 3 | 4.4 (2.1, 8.2) |
| **12** | 108 | 12 | 6 | 16.1 (10.7, 22.3) | 115 | 15 | 1 | 6.6 (3.5, 11.2) |
| **13** | 90 | 6 | 8 | 21.1(14.8, 28.1) | 99 | 6 | 0 | 7.5 (4.0, 12.4) |
| **14** | 76 | 9 | 4 | 28.3 (20.8, 36.3) | 93 | 14 | 1 | 7.5 (4.0, 12.4) |
| **15** | 63 | 4 | 3 | 32.3 (24.2, 40.6) | 78 | 8 | 1 | 8.6 (4.7, 13.9) |
| **16** | 56 | 5 | 2 | 35.6 (27.0, 44.2) | 69 | 10 | 1 | 9.7 (5.4, 15.8) |
| **17** | 49 | 17 | 3 | 37.9 (29.0, 46.8) | 58 | 24 | 1 | 11.1 (6.2, 17.6) |
| **18** | 29 | 5 | 1 | 42.3 (32.6, 51.7) | 33 | 8 | 2 | 13.4 (7.3, 21.5) |
| **19** | 23 | 6 | 1 | 44.5 (34.1, 54.4) | 23 | 6 | 1 | 19.1 (10.1, 30.3) |
| **20** | 16 | 4 | 0 | 46.9 (35.8, 57.3) | 16 | 5 | 0 | 23.9 (12.0, 38.1) |
| **21** | 12 | 3 | 1 | 46.9 (35.8, 57.3) | 11 | 2 | 1 | 23.9 (12.0, 38.1) |
| **22** | 8 | 1 | 0 | 52.8 (36.9, 66.5) | 8 | 1 | 0 | 32.4 (13.9, 52.5) |
| **23** | 7 | 2 | 1 | 52.8 (36.9, 66.5) | 7 | 2 | 1 | 32.4 (13.9, 52.5) |
| **24** | 4 | 1 | 0 | 62.3 (36.7, 79.9) | 4 | 1 | 0 | 43.6 (16.6, 68.1) |
| **25** | 3 | 3 | 0 | 62.3 (36.7, 79.9) | 3 | 3 | 0 | 43.6 (16.6, 68.1) |

[a] cumulative incidence (%) was estimated by product limit (Kaplan-Meier) survival probability

[b] Includes: Thyroiditis, Psoriasis, Inflammatory bowel disease (IBD), Celiac disease, Behcet's disease, Systemic lupus erythematosus (SLE), Diabetes mellitus Type I; does not include arthritis or PANS

[c] Participants only contribute person-time until they have developed the condition of interest. Once they develop the health outcome of interest (i.e., arthritis or other autoimmune disease) they drop out of being in the denominator. The denominators for each year are different between two incidence tables as cases that develop arthritis each year is a different number than cases that develop an autoimmune disease each year.

© 2024 Ma M et al. *JAMA Network Open.*

**eTable 2.** Incidence Rates for Arthritis and Autoimmune Disease Among Consecutive Patients With PANS, N=193

| | | juvenile-onset arthritis (ILAR and/or ASAS criteria) | | | other autoimmune diseases[a] | | |
|---|---|---|---|---|---|---|---|
| **Variables** | **Patients, no.** | **Person-years, no.** | **Cases, no.** | **IR per 100,000 person-years** | **Person-years, no.** | **Cases, no.** | **IR per 100,000 person-years** |
| **Overall** | 193 | 2,595 | 55 | 2,120 | 2,662 | 21 | 789 |
| | | | | | | | |
| **Sex** | | | | | | | |
| male | 112 | 1,570 | 34 | 2,166 | 1,638 | 9 | 550 |
| female | 81 | 1,025 | 21 | 2,049 | 1,023 | 12 | 1,173 |

Abbreviations: IR- incidence rate

[a] Includes: Thyroiditis, Psoriasis, Inflammatory bowel disease (IBD), Celiac disease, Behcet's disease, Systemic lupus erythematosus (SLE), Diabetes mellitus Type I; does not include arthritis or PANS

© 2024 Ma M et al. *JAMA Network Open.*
