# Supplementary material for: Development of Autoimmune Diseases Among Children With Pediatric Acute-Onset Neuropsychiatric Syndrome
Source: JAMA Netw Open. 2024 Jul 30;7(7):e2421688. doi: 10.1001/jamanetworkopen.2024.21688 (PMC11289697; doi:10.1001/jamanetworkopen.2024.21688)
Supplement: Supplement 2. — Data Sharing Statement [file jamanetwopen-e2421688-s002.pdf]

Data Sharing Statement

Ma. Development of Autoimmune Diseases Among Children With Pediatric Acute-Onset Neuropsychiatric Syndrome. *JAMA Netw Open*. Published July 30, 2024. doi:10.1001/jamanetworkopen.2024.21688

Data
Data available: Yes
Data types: Deidentified participant data
How to access data: All de-identified participant data used in these analyses and related documents (study protocol, statistical analysis plan) will be shared on reasonable request from any qualified investigator via email to meima@stanford.edu or jfranko@stanford.edu
When available: With publication

Supporting Documents
Document types: Statistical/analytic code
How to access documents: Will be shared on reasonable request from any qualified investigator via email to meima@stanford.edu or jfranko@stanford.edu
When available: With publication

Additional Information
Who can access the data: on reasonable request from any qualified investigator
Types of analyses: for specified purpose
Mechanisms of data availability: after approval of a proposal or with a signed data access agreement
